# Towards Clinically Useful Quantitative Lymphoscintigraphy: A Scoping Review

**DOI:** 10.3390/lymphatics4020020

**Published:** 2026-04-14

**Authors:** Juliana H. Kim, Sina Oh, Alex Heglin, Jaewon Yang, Orhan K. Öz, Robert C. Sibley

**Affiliations:** Department of Radiology, University of Texas Southwestern Medical Center, 5323 Harry Hines Blvd., Dallas, TX 75390-9069, USA

**Keywords:** lymphedema, lymphatic imaging, quantitative, lymphoscintigraphy

## Abstract

Lymphedema is a chronic condition marked by swelling in the body’s soft tissues due to impaired or damaged lymphatic function. Lymphedema is estimated to affect about 1 in 1000 in the United States and 1.4 per 1000 based on two European studies. Lymphedema diagnosis relies on clinical visual assessment by physicians, and the quantitative evaluation of lymphatic function is not widely employed due to the lack of standardized and validated metrics. Lymphoscintigraphy is considered the practical gold standard for evaluating lymphedema. With the emergence of advanced surgical therapies, there is a growing demand for quantitative evaluation metrics to objectively evaluate treatment response. To address this unmet need, we reviewed various methods of quantitative lymphoscintigraphy (qLSG) that were used for assessing lymphedema. We found that multiple qLSG approaches have been described. Moreover, the protocols vary widely in terms of tracer type, injection route, dosage, and exercise interventions. We compared the quantitative assessment strategies and highlighted the approaches that may help provide a more objective lymphedema diagnosis and follow-up.

## 1. Introduction

Lymphedema remains a poorly studied disease despite its common occurrence and chronicity. The exact prevalence is unknown. It is estimated to affect about 1 in 1000 in the United States and 1.4 per 1000 based on two European studies [1]. However, this is likely a gross underestimate, as lymphedema often remains unreported, especially in its early stages [2]. The lack of documented disease is likely explained by the poor ability to quantify subtle lymphatic dysfunction and the underutilization of limited guidelines for staging and treatment.

Sherman and Ter-Pogossian introduced the method of lymphoscintigraphy (LSG) in 1953, offering insight into the function of the invisible lymphatic system. The initial purpose of quantification was to use LSG for cancer evaluation, rather than for diagnosing the dysfunction of the lymphatic system. From the beginning, the strength of LSG has been that radioactive molecules intravasate from the injected site into the lymphatic system and can be easily detected by gamma cameras. It is worth noting that LSG protocols typically include 2D planar imaging, which does not represent quantitative imaging, i.e., the pixel values are not absolute activities (e.g., Bq/mL) but measured counts (e.g., counts per second). Currently, physicians’ visual assessments remain the clinical standard for evaluating lymphedema. In the broader nuclear medicine literature, “quantitative” imaging generally refers to an estimation of absolute activity concentration using physical corrections such as attenuation correction. However, because conventional 2D planar lymphoscintigraphy does not generally support such correction frameworks, it is not fully quantitative in this strict sense. Accordingly, in this review, we use the term “quantitative” to refer to the quantitative analysis of LSG findings beyond a purely visual interpretation. Though LSG allows for quantitative evaluation metrics using measured counts, quantitative LSG (qLSG) has not been adopted in clinical practice in part because the benefits of using qLSG have not been standardized and clinically validated. Importantly, this does not reflect an inherent technical limitation of qLSG but rather the lack of widely accepted protocols and analysis techniques, which have resulted in poor reproducibility and substantial interstudy variability, preventing the accumulation of consistent clinical evidence. Now, as the number of lymphatic diseases and therapies for lymphatic intervention is growing, the need for qLSG is emerging [3–5]. qLSG has attracted clinical interest because of its potential to detect early or subtle lymphatic functional abnormalities that may be missed by qualitative lymphoscintigraphy, thereby contributing to early diagnosis and disease staging. It is worth noting that “lymphatic function” is not a single physiologic construct, and different qLSG metrics may reflect distinct aspects of lymphatic biology and dysfunction. For example, the nodal uptake percentage assesses the ability of the lymphatic system to take up the tracer through the lymphatic capillary network, pump the tracer through lymphatic vessels, and for the tracer to accumulate in lymph nodes. However, this test may be inadequate in patients who have had a removal of lymph nodes or who have had a lymphovenous bypass with a tracer bypassing the nodal basin. The quantitative metrics that rely on placing regions of interest (ROIs) over the skin will detect the retrograde flow from the collecting lymphatic vessels into the dermal lymphatics. This type of metric assesses a certain type of lymphatic pathology known as dermal backflow. A dermal backflow in lymphoscintigraphy is pathognomonic for lymphedema.

Though no quantitative evaluation techniques for LSG have been translated to routine clinic practice, many approaches have been described in the literature. Here, we reviewed the use of qLSG to evaluate patients with lymphedema, summarizing the variety of protocols used, measurements obtained, and potential clinical applications. The results demonstrate the lack of continuous improvement and failure of qLSG to translate broadly to clinical practice. Finally, we discuss the opportunities to further standardize protocols and apply qLSG to clinical practice.

Although newer techniques, including magnetic resonance lymphangiography (MRL), indocyanine green (ICG) near-infrared (NIR) imaging, and high-frequency ultrasound, have increasingly been used to evaluate patients with lymphatic disease, LSG is still referred to as the reference standard in the evaluation of lymphedema due to its high sensitivity (96%) and specificity (100%) [6]. In 2023, the Society of Nuclear Medicine and Molecular Imaging (SNMMI) gathered an expert workgroup to evaluate the appropriate use criteria for LSG. Within the clinical domain of lymphedema, they found that LSG remained highly appropriate for evaluating primary lymphedema, limb edema of unclear etiology, and secondary lymphedema. They noted that LSG is particularly helpful in differentiating edema caused by veno-occlusion from that caused by lymphatic blockage [7].

The lack of standardized LSG protocols is the primary barrier to the usefulness of qLSG [7–10], though, in general, other limitations of LSG include the long duration of studies and radiation exposure to patients [11]. Though LSG is the reference standard, the suboptimal protocols and analysis decrease the diagnostic utility of LSG. For example, recent studies suggesting the inferiority of LSG to ICG NIR imaging highlighted the impact of suboptimal LSG protocols on the study’s effectiveness. In 2012 Mihara et al. demonstrated that LSG had a 62% sensitivity for upper arm lymphedema versus a 100% sensitivity using ICG NIR in a study of 21 cases, where the sensitivity of lymphedema diagnosis was impacted by the imaging protocol (mild massage but no exercise of the arms) as well as the evaluation metric (presence of dermal backflow only) [11]. Similarly, in 2020, Figueroa et al. reported that LSG had a sensitivity of 54% compared to ICG, which had a 88% sensitivity in a study of 50 cases where LSG studies were done at outside institutions under varying protocols: no specifications about timing, post-injection exercise, or method of injection (subcutaneous vs. intradermal) were provided in the LSG studies [8].

As a result of the lack of standardized protocols and quantitative evaluation metrics, there is a limited consensus about diagnosis criteria, staging, or treatment follow-up methods for lymphedema [7,10]. The Genoa protocol was developed in 2019 to provide procedural recommendations for LSG. However, the method relied on ^99m^Tc-Nanocoll, which is not available in the United States. The primary measurement used was the transport index, which is a semi-quantitative method that still relies on subjective interpretation and may involve a comparison with the presumed unaffected side of the body.

To facilitate the development of a standardized qLSG approach, we performed a systematic scoping review of qLSG methods used to quantitatively evaluate lymphedema. We aimed to provide an overview of qLSG so that it can be properly used for objective evaluation alongside visual assessment and can be developed toward therapeutic uses.

## 2. Methods

### 2.1. Article Search Strategy

We adhered to the Preferred Reporting Items for Systematic reviews and Meta-Analyses extension for Scoping Reviews (PRISMA-ScR) guidelines. The protocol may be obtained by contacting the principal author. The PRISMA-ScR checklist is available in Supplementary Table S1 [12]. To search for qLSG-related publications, PubMed and Ovid Medline were used. Our search included studies that met three domain criteria: (1) lymphoscintigraphy, (2) edema or lymphedema, and (3) quantitative, clearance, rate, uptake, depth correction, or activity curves. The Medical Subject Headings (MeSH) terms that were used in the search included lymphoscintigraphy, edema, lymphedema, lymphoedema, quantitative, clearance, rate, uptake, depth, correction, activity, and curve.

### 2.2. Eligibility Criteria

The articles were included in the review if they met the following criteria: (a) any peer-reviewed article that presented data, such as original articles, short communications, and research letters; (b) articles that reported quantitative measurements taken from lymphoscintigraphy; and (c) lymphoscintigraphy that was performed in human patients who were evaluated for lymphedema. The articles with the following criteria were excluded: (a) articles that did not report original data, such as reviews, systematic reviews, and meta-analyses; (b) case reports or case series that included less than 10 patients; (c) articles that were not written in English; (d) articles that only reported semi-quantitative measurements (i.e., transport indices), and (e) articles that did not assess lymphedema.

### 2.3. Article Selection and Collection

PubMed and Ovid filters were used to exclude meta-analyses, review articles, and case reports. The final search results were exported into Covidence systemic review software (Veritas Health Innovation, Melbourne, Australia, available at www.covidence.org), and the duplicates were removed. The articles were independently screened by JK and RS. The articles were initially screened for relevance based on their titles and abstracts, according to the eligibility criteria. The articles deemed irrelevant during the initial screening by total agreement between JK and RS were removed. The remaining articles proceeded to the full-text review, where the authors categorized the reasons for their study exclusion in-depth. Cohen’s Kappa score was calculated to be 0.47, which showed a moderate agreement between the assessor pairs [13]. Disagreements that occurred in the full-text review were resolved through discussion and consensus. The extracted information included the title, authors, publication year, DOI, PubMed ID, funding sources, radiotracer, filtration status of the tracer, particle size of the tracer, volume of injection, radiation dose per injection, route of injection, regions of interest, exercise interventions, quantitative measurements reported, timing of imaging acquisition, and the major conclusions drawn about qLSG. Table 1 shows the summary statistics for the publication year, radiotracer used, filtration status, particle size, volume of injection, radiation dose per injection, route of injection, exercise interventions, quantitative measurements reported, and total duration of the study. In Table 1, “Unknown” indicates the information that was not reported in the original study.

## 3. Results

### 3.1. Results of the Search

Out of 191 unique studies imported for screening, 68 studies were deemed irrelevant during the initial screening and were excluded, and 123 moved on to full-text review. Of those, 71 met the eligibility criteria. The most common reason for exclusion during the full-text review was that the results of the LSG studies were not quantitative (47%) or were only semi-quantitative (20%). Eight articles were case reports, case series, or review articles that were not excluded by the database filters. Seven articles were excluded because we did not have full-text access. Six articles were written in a language other than English. One article focused on magnetic resonance lymphangiography without reporting the results of lymphoscintigraphy (wrong intervention). Figure 1 shows the workflow of selecting the articles for full-text review. The study’s country of origin, LSG protocols, and quantitative measures are presented in Table 1.

### 3.2. Radiotracers

Nine different radiotracers were identified. Among the 71 quantitative studies, the most common radiotracer used was the ^99m^Tc-human serum albumin (28/71 or 39%), followed by the ^99m^Tc-human immunoglobulin (14/71 or 20%), and the ^99m^Tc-phytate (11/71 or 15%). There were several studies that used multiple tracers. The variation in radiotracers is expected, given the many nationalities represented among the studies and the differences in the tracers that are approved in each country. The studies included in our review took place in seventeen countries; most of the studies occurred in the United Kingdom (26/71 or 37%), followed by South Korea (9/71 or 13%). The majority of studies did not mention whether the radiotracer was filtered (47/71). The lymphatic-specific radiotracers (e.g., ^99m^Tc sulfur colloid) were typically filtered through 100 or 200 nm filters to produce particles in the range of 15–100 nm [85]. Unfiltered colloids may contain large particles in the hundreds of nanometers (e.g., ~200–300 nm or more) [86], which results in extremely slow transport and prolonged scan durations. An even greater number did not specify the particle size (59/71), making the particle size and filtration status the most commonly missing protocol elements in Table 1.

### 3.3. Injection Protocols

The volume of each tracer injection was described in most studies (49/70). The mean and median injection volumes were 0.26 mL and 0.20 mL, respectively. If a range was given, the average of the minimum and maximum was taken. If a threshold was listed, the maximum was used. The mean and median doses of radiation per injection were 46 MBq (1.24 mCi) and 35 MBq (0.95 mCi), respectively. If multiple dosages were described, we took the average of the doses. The most common route of injection was subcutaneous (54/71 or 76%). Intradermal (intracutaneous) injections were used in 14/71 or 20%. Four studies included multiple routes of injection in their protocols. In general, the radiotracer is administered subcutaneously or intradermally at the first web space of the dorsal surface of each foot or hand.

### 3.4. Exercise Interventions

About half of the studies did not include any standardized exercise intervention in their protocol (49%). Among the studies that included exercise, hand exercises were the most common (12/71). The hand exercises included grip and release and squeezing a ball for 5–20 min, or until a specified number of exercises were completed. Some protocols specified the frequency of the hand squeezing by using a metronome [30,58].

Seven studies (7/71 or 10%) used walking as an exercise, requiring patients to walk anywhere from 20 to 60 min, either at their natural pace, at a “brisk pace,” or using equipment such as the treadmill to standardize the speed (e.g., 3.2 km/hour) or a mini stepper to standardize the number of steps (e.g., 20 times) [23,50]. Applying pressure to the extremity or massaging the site of injection alone was performed in two studies. Seven studies included multiple forms of exercise in their protocols. Often, these studies combined the upper and lower extremity exercise or included both the massage and the active movements. Some protocols specified rest periods before the tracer injection. The benefits of the exercise interventions include increasing the amount of radiotracer uptake by the lymphatic system and allowing a shorter study duration [10]. However, exercise interventions may compromise reproducibility and reduce the feasibility in routine clinical practice due to increased protocol complexity and the varying ability of patients to perform certain exercise protocols.

### 3.5. Imaging Protocols

The duration of the studies from the time of injection to the last image acquisition ranged from two minutes to six hours. The mean duration was 152 min. Most protocols only used static acquisitions (45/71), while 20/71 included both static and dynamic acquisitions; the dynamic portion was usually performed immediately after injection but sometimes was started immediately after exercise.

In 2019, Villa et al. proposed the Genoa protocol to standardize lymphoscintigraphy protocols. They argued that both dynamic and static acquisitions were necessary; the dynamic acquisition was needed to determine the lymphatic flow, and the static acquisition was needed to capture the dermal backflow. Other recommendations included performing both subfascial and epifascial injections in order to detect abnormalities of the deep lymphatic system and using SPECT/CT when the disease was located in the pelvic/abdominal/thoracic regions. However, their recommendations were not widely adaptable because they relied on the use of ^99m^Tc-Nanocoll, which is available in Europe but not in the USA. Furthermore, following all the recommendations is cumbersome, as the two-compartment study would require 48 h to allow for a washout period in between.

Another effort towards standardization was completed by Pappalardo et al. in 2020 [10]. Their recommended protocol used ^99m^Tc-phytate and combined dynamic and static imaging over two hours. They developed the Taiwan lymphoscintigraphy staging (TLS) system to improve upon the International Society of Lymphology (ISL) staging system, which is only based on clinical signs and has been shown to have no correlation with LSG findings. While they demonstrated correlation with clinical outcomes, their staging system solely relied on qualitative findings, which can be difficult to interpret.

### 3.6. Regions of Interest

The injection site was the most common region of interest (ROI) drawn to make quantitative measurements (46/71 or 65%). The measured counts from the injection site were used to measure clearance. The initial counts at time zero were also used as the denominator of the uptake percentage. The measured counts at the lymph nodes (LN) were commonly included as ROIs to quantitatively evaluate lymphedema (61/71), with 30/71 studies including the inguinal LN as an ROI to evaluate lower extremity lymphedema, and 27/71 studies including the axillary LN to evaluate the upper extremity. The ROIs were drawn over the lymphatic vessels above or below LNs in 35/71 studies. A few studies included the liver as an ROI to assess the transit of lymph flow into the blood pool.

### 3.7. Quantitative Measurements

At least 18 different quantitative measures were employed (Table 2). These metrics are not necessarily interchangeable because they reflect different aspects of lymphatic function. For example, nodal uptake-based measures primarily reflect the tracer delivery to and accumulation within the regional lymph nodes. The clearance-based measures reflect tracer removal from the injection depot and transport from the interstitium into the lymphatic system. The transport-related measures, such as appearance time or mean transit time, reflect the timing and efficiency of lymphatic transit. The dermal backflow-based measures reflect the abnormal retrograde lymphatic flow patterns rather than the nodal accumulation or depot clearance.

#### 3.7.1. Uptake Percentage

The most common measurement was the *uptake percentage*, which is a ratio between the counts at an ROI (corrected for physical decay) divided by the initial count at the injection site. The frequently used ROIs included the lymph nodes of the axilla or inguinal regions and the lymphatic vessels of the upper or lower extremity (Table 1). Various thresholds have been proposed to assess disease severity; however, these values depend on the radiotracer and the exercise protocol used [50,87]. This measurement was reported in 37/71 or 52% of the studies.

#### 3.7.2. Clearance Rate

The second most common quantitation was the *clearance rate (rate constant) k*, which is determined from the slope of the linear phase of a time-activity curve (used by 24/71 of the studies or 34%). Calculating the clearance rate involves plotting the logarithm of the fraction of the counts remaining in the depot (after correction for radionuclide decay) vs. time. The slope of the linear plot gives the fraction of tracer removed per minute. This value reports the steady state flow of interstitial fluid into the lymphatic system. Therefore, it is independent of the injected volume of the tracer when the volume exceeds a threshold [88]. On the other hand, the value is dependent on which area of the lymphatic vessel is selected as an ROI. The clearance rate may aid the lymphedema diagnosis, but it correlates poorly with clinical severity. Finally, the clearance rate is affected by low radiochemical purity and geometrical changes in the depot (i.e., if activity moves outside the field of view of the detector) [18,88]. The counterpart to the clearance rate is the *uptake rate*, which is the slope of the linear plot of counts accumulated vs. time for a given ROI. This calculation will require a longer study if lymphatic function is abnormal, and it was only reported in three studies [38,58,66].

#### 3.7.3. Uptake Ratio

The *uptake ratio* is calculated as any fraction comparing counts between two ROIs at a single point in time. The uptake ratio comparisons were used in 12 studies. The most common ratio compares the measured counts of the affected and non-affected sides (quantitative asymmetry index (QAI). The QAI is dependent on the contralateral side being healthy and may not correlate with the severity of lymphedema, depending on the ROI chosen [78]. Another type of *uptake ratio* compares the maximum counts over a linear lymphatic vessel and the mean count of a background ROI consisting of one flank region (without the kidney, ureter, and bladder) [82]. A third type of ratio compares the superficial and deep lymphatic counts [72]. Other ratios compare the maximum counts before and after exercise intervention (i.e., relative increase in maximal activity) [31]. By contrast, the *lymphatic drainage efficiency*, introduced by Keramida et al., compares the uptake in the ilioinguinal nodes with the counts remaining in the injection depot at a specific time [71].

#### 3.7.4. Clearance Percentage

The *clearance percentage* is the counterpart to the *uptake percentage* mentioned previously and measures the ratio between the count at the injection site at a specified time, which is then divided by the initial count. This measurement can be used to assess the global lymphatic function in patients who have undergone lymphatic procedures, such as lymphadenectomy or radiotherapy, in contrast to the uptake percentage [29]. The clearance percentage was measured in nine studies.

#### 3.7.5. Appearance Time

This is the time taken to reach either the background activity or half the peak activity and is also referred to as the *transit time*. Dabrowski et al. noted that this measurement is highly variable depending on the tracer used, its volume, its pH, its electrical charge, and its hydrophilicity [48]. This measurement was reported in 8/71 or 11% of the studies. Similarly, the *half clearance time* can be calculated as the time until half of the initial uptake recorded at the injection depot is removed (reported in 5/71 or 7% of the studies). Weissleder et al. demonstrated that quantitative parameters, such as the *appearance time*, were necessary to detect early-stage primary lymphedema. They found mild lymphedema in 48 of 73 extremities through analyzing the quantitative parameters, though the qualitative LSG appeared normal. However, the quantitative dynamic measurements after passive and maximal exercise demonstrated abnormalities in lymphatic function [17].

#### 3.7.6. Uptake Counts per Time

This is simply the counts measured within an ROI or per unit volume during a single time point (i.e., the counts obtained during a one-second acquisition). The counts were often corrected for physical decay by dividing the measurements by the initial counts measured at the time of injection. Alternatively, the counts were normalized by background subtraction. The studies either reported counts per second or per minute [18,20,24,29,45]. These values cannot be easily compared across the studies, since they do not account for varying doses, patient metrics, timing, or system calibration.

#### 3.7.7. Rate Constant Ratio

The rate constant ratio compares the rate constants of two ROIs (i.e., affected and unaffected sides; subcutis and muscle). This was reported in 7/71 of the studies.

#### 3.7.8. Blood Appearance Rate

This is measured by a linear constant, which was measured in seven studies, usually by venous sampling. The calculation involves taking a blood sample from the unaffected, contralateral arm. The sample concentration (in units of percent dose per liter) is multiplied by the patient’s blood volume (in liters, estimated from height, weight, sex, and age using a standard equation). This measurement is dependent on the percentage of protein-bound radiotracer, which varies depending on the agent [39]. Alternatively, one study used the counts at the liver and heart to determine the constant [37]. A related measurement is the *blood appearance percentage*, which is the percentage of initially administered activity found per liter of blood (2/71).

#### 3.7.9. Mean Transit Time (MTT)

The *mean transit time* (MTT) is described as a measurement that is independent of the radiotracer used, the injection route, and the dose [70]. The MTT was developed by Hvidsten et al. in 2018 to quantitatively distinguish normal lymphatic function from pathologic lymphatic dysfunction [70]. The MTT calculation is similar to the MTT found in other nuclear medicine studies (e.g., the esophageal or gastric emptying MTT), where MTT is calculated by the area under the time activity curve divided by the height [89]. To deal with limited acquisition times, they described a time activity curve model that could be generated by combining two quantitative measurements: the *clearance rate constant* and the *uptake rate constant*. The MTT does not require a comparison with an unaffected side of the body, providing benefits over the other quantitative analysis techniques described. However, drawbacks include that the MTT is not reliable when the tracer has limited migration from the injection depot. Also, if the tracer is slow or lymphatic flow is delayed, a decline in counts may not be detected, preventing the calculation of the MTT.

#### 3.7.10. Model Fitting

Hvidsten et al. developed best-fit models of the time-activity curves to derive model constants, which were then compared between the patients. The study duration lasted five hours, with images acquired every 30–45 min to generate enough time points to create a model [70]. Gebousky et al. introduced the Bayesian approach to allow modeling based on a limited number of time points (3–4 points). The model assumed that tracer appearance in an ROI starts after a delay, follows an exponential rise, and reaches a plateau proportional to gain. Applying the Bayesian theory, the model incorporates population-level data to create realistic models for each patient [53]. By contrast, Baulieu et al. proposed *factorial analysis* as an unsupervised method to decompose the spatial and temporal data from a dynamic acquisition study into a few factors. Each of the factors was interpreted to correspond to a physiological phase. For example, a pattern with a rapid rise in activity corresponds to the rapid tracer migration from the injection site to the first part of the arm. Another pattern with a slower rise in activity may correspond to migration proximally further up the arm. Lastly, a pattern of stable activity likely represents the minimal diffusion that occurs at the injection site [18].

#### 3.7.11. Contrast-to-Noise Ratio (CNR) of DBF

The contrast-to-noise ratio was introduced by Koike et al. to quantify the dermal backflow for the SPECT-CT data. The mean background count is subtracted from the mean count of the area of dermal backflow (DBF); the result is divided by the standard deviation of the background counts. Weaknesses include that the DBF is dependent on the tracer used and does not show significant differences between the groups of varying clinical severity and chronicity [82].

### 3.8. Funding Sources

A large portion of the studies did not state whether funding was given (32/71). Of those studies that specified a funding source (26/71), the Wellcome Trust was the most common contributor (11/71). Eight studies were at least partially funded by government grants.

## 4. Discussion

Due to the difficulty in reproducing quantitative measurements and the many variations in protocols, qLSG has limitations compared to qualitative LSG [10,49,56,81]. Quantitative LSG is often viewed as an adjunct study and is not routinely performed, despite its utility in diagnosing early-stage lymphedema and distinguishing lymphedema from extra-lymphatic causes of edema. We found that only 53% of the studies evaluating lymphedema employed quantitative methodology, and only 20% used semi-quantitative methods.

The most common quantification was the *uptake percentage*, which is tracer- and protocol-dependent. This technique offers thresholds that differentiate disease severity. However, in isolation, the nodal uptake percentage has several limitations. Modi et al. recognized that the measurements based on the lymph node activity alone are problematic because (1) the node numbers vary between people, (2) the node numbers can be decreased in patients who undergo a lymphadenectomy, and (3) there is no agreed-upon normal range of nodal uptake percentages. Additionally, the nodal uptake percentage is a limited assessment strategy in patients who are treated with lymphatic venous anastomosis, as the treatment bypasses the nodal basin.

The second most common quantitation, the *clearance rate (rate constant)*, was developed to mitigate the weaknesses of the uptake percentage. Modi et al. proposed using the clearance rate *k* and showed that this value could distinguish intrinsic lymphatic dysfunction from other causes of edema. Lymphedema caused a decrease in *k*, while *k* increased in the setting of exercise, hyperfiltration edemas (e.g., cardiac edema, hypoproteinemic edema), and inflammation [88]. We found that only 35% of the studies overall and 19% of the qLSG studies published after the introduction of the clearance rate constant *k* incorporated this value into their analysis. The poor reception of the *clearance rate* may be due to Jensen et al.’s demonstration that it could not reliably distinguish lower extremity lymphedema from healthy controls. They hypothesized that their poor results were due to protocol variations, namely, their use of ^99m^Tc-HSA, which has a smaller particle size compared to ^99m^Tc-Nanocoll. They concluded that capturing the *rate constant k* was impractical because it depended on many factors, such as the size of the radiotracer, the route of the injection, the injection volume, and the particle concentration [56].

Beyond a frequency-based summary, we reviewed the two most frequently used metrics, the *uptake percentage* and the *clearance rate*, from a clinical perspective. The *Uptake percentage* showed the strongest support for early-stage detection and somewhat broader support for the correlation with clinical severity. The *clearance rate* also showed their main strength in early-stage detection, but their association with clinical severity was less consistent. For both metric groups, the studies on treatment response were limited. The studies on reproducibility and standardization were also limited and were mainly focused on method development or feasibility. Overall, the most supportive studies were classified as Level 3b or Level 4 by the Oxford Center for Evidence-Based Medicine Levels of Evidence criteria [90].

Although most quantitative measurements cannot be compared across studies with different protocols, we noted several exceptions. The *uptake ratios* comparing affected and unaffected sides of the body serve as internal controls, allowing patients to be compared with one another. Examples include the *quantitative asymmetry index (QAI)* and the *clearance/uptake rate ratios* comparing affected and normal limbs. However, they require that the patients have unilateral lymphatic disease. The *count ratios* comparing pre- and post-exercise measurements also partially account for variation, as long as the exercise intervention is standardized. Furthermore, they do not require a normal limb. Other promising qLSG methods include creating convolution or deconvolution models of the time-activity curves, then identifying population-based indices of normal *appearance times*, *clearance rates*, or *mean transit times*. Convolution models, such as Bayesian qLSG, are useful for static studies with few time points. Deconvolution models, such as dynamic factorial analysis, will be better for data-rich dynamic studies performed in a research environment.

Out of the 68 studies we were able to access, we found only three protocols that were repeated at least once when considering the radiotracer used, the injection route, the exercise intervention, and the image acquisition protocol. The lack of standardized lymphoscintigraphy quantitation has limited the diagnosis, treatment, and follow-up of lymphedema. Since primary and secondary lymphedema remain difficult to distinguish in patients, standardized qLSG is also needed to identify those who have pre-existing defects in their lymphatic system prior to a secondary insult [91]. To standardize protocols, Tartaglione et al. argued that the exercise protocols with intradermal injections facilitated lymphoscintigraphy. Compared to the rest of the protocols with subcutaneous injections, the exercise intradermal protocols significantly shortened the examination times (20 min vs. several hours) and minimized the interference of the background activity and the extravasation of the injected tracer [92]. However, this method still did not take hold: it was never repeated, and only three subsequent studies used exercise intradermal protocols, highlighting the need for greater collaboration and consensus among lymphatic imaging specialists.

The limitations of this review are that the studies written in a language other than English were excluded (*n* = 6). Given the many variables involved in LSG, it is impossible to categorize every aspect of the protocol across the diverse patient populations and regulatory environments. Due to the wide variation in methods, we grouped similar calculations as a single type of quantitative measurement. For example, the uptake ratio included the quantitative asymmetry index as well as the ratios between the counts of the superficial and deep lymphatic systems.

## 5. Conclusions

Our review demonstrated that many combinations of radiotracers, injection methods, exercise interventions, and image acquisition timing protocols have been employed to enable quantitative LSG (qLSG) for objective lymphedema evaluation. Since the LSG protocols varied across the countries where LSG was performed, the resources available, and the patient population, it is challenging to develop a one-size-fits-all LSG protocol. However, qLSG may enable a quantitative measurement of lymphedema that could be compared across studies, which can improve the diagnosis, treatment, and understanding of the disease. This study highlights the need for qLSG that can provide quantitative analysis metrics for objective evaluation in patients with lymphedema. Further studies are needed to establish their clinical usefulness and reliability.

## Supplementary Material

supplementary material

**Supplementary Materials:** The following supporting information can be downloaded at https://www.mdpi.com/article/10.3390/lymphatics4020020/s1. Table S1: Preferred Reporting Items for Systematic reviews and Meta-Analyses extension for Scoping Reviews (PRISMA-ScR) Checklist.

## Figures and Tables

**Figure 1.**
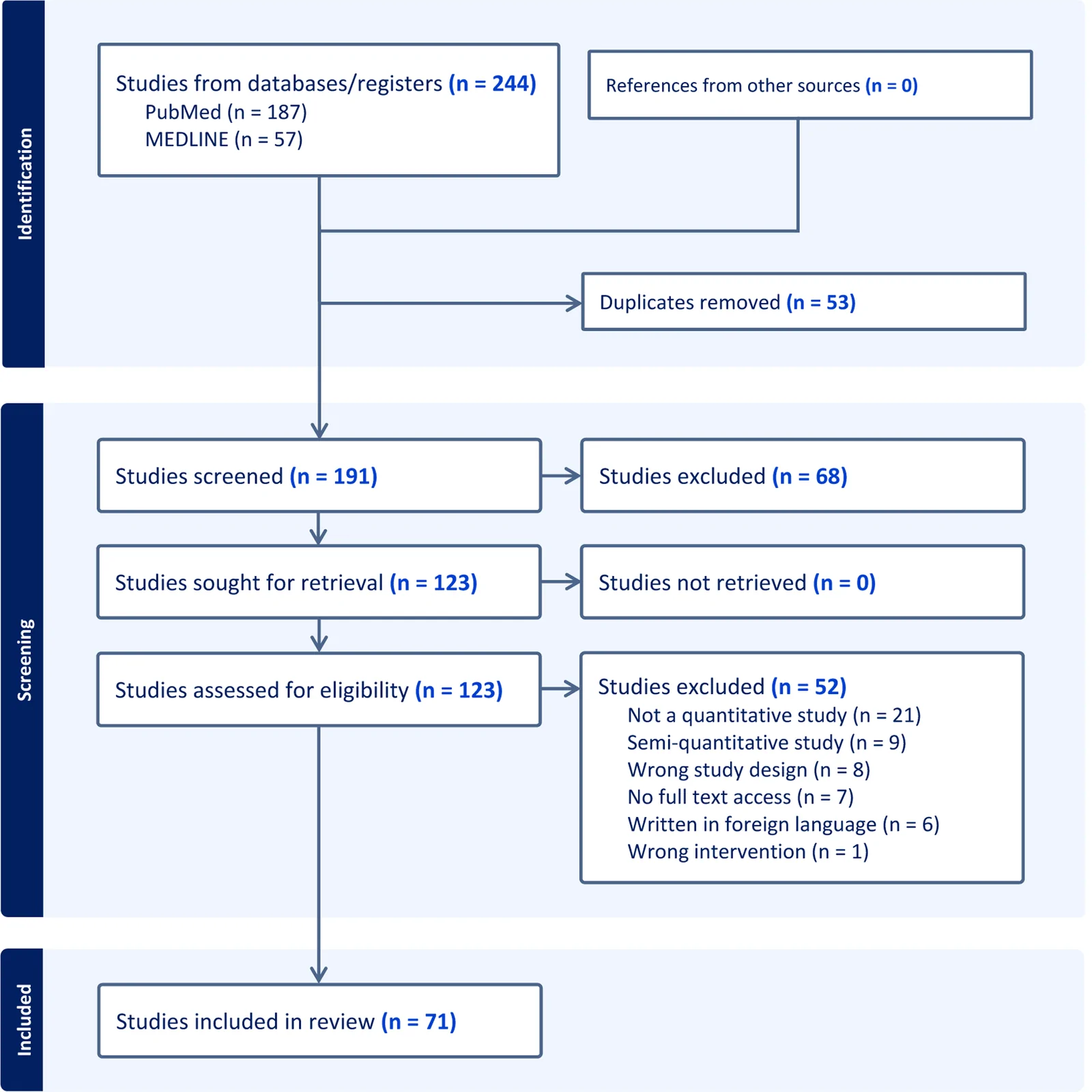
The PRISMA flow diagram for scoping reviews.

**Table 1.** The study protocols involving quantitative lymphoscintigraphy.

Year	First Author	Radiotracer	Filtered?	Particle Size	Volume (ml)	Radiation Per Injection (MBq)	Route ^^^	ROI	Exercise	Measurement	Duration * (s)	Acquisition Type	Country	Reference
1985	Stewart G	^99m^Tc-Rhenium sulfide	Unknown	Unknown	0.25	75	sc	IS, inguinal LN	None	Uptake percentage	180	Both	UK	[14]
1986	Vaqueiro M	^99m^Tc-sulfur colloid	Unknown	Unknown	Unknown	18.5	sc	IS, inguinal LN, axillary LN, liver	Multiple	Uptake percentage	180	Static	USA	[15]
1988	Carena M	^99m^Tc-sulfur colloid	No	<50 nm	0.1–0.2	18–20	sc	IS, inguinal LN, axillary LN, lower ext, upper ext	None	Uptake percentage and clearance rate	360	Static	Italy	[16]
1988	Weissleder H	^99m^Tc-HSA	No	<80 nm	0.25 ± 0.05	37	sc, im	IS, inguinal LN, axillary LN, lower ext	Multiple	Uptake percentage, uptake counts per second, and appearance time	120	Both	Germany; USA	[17]
1989	Baulieu F	^99m^Tc-sulfur colloid	Unknown	Unknown	Unknown	111	sc	IS, inguinal LN, lower ext	Multiple	Uptake counts per minute and factorial analysis	240	Both	France	[18]
1989	Baulieu F	^99m^Tc-Rhenium sulfide	Unknown	Unknown	Unknown	111	sc	IS, lower ext, upper ext	Pressure	Uptake counts per minute, clearance rate ratio, and uptake rate ratio	240	Both	France	[19]
1990	Rijke AM	^99m^Tc-sulfur microcolloid	No	<50 nm	0.1	111	sc	IS, inguinal LN, lower ext	Multiple	Uptake counts per minute and clearance rate (rate constant)	180	Static	USA; Netherlands	[20]
1993	Bräutigam P	^99m^Tc-HSA	Unknown	Unknown	Unknown	30	sc	inguinal LN	Multiple	Uptake percentage	120	Both	Germany	[21]
1993	Bull RH	^99m^Tc-antimony sulfide	Unknown	Unknown	0.1	20	sc	IS, inguinal LN	Walking	Uptake percentage	120	Static	UK	[22]
1993	Liu NF	^99m^Tc-HSA	Yes	Unknown	0.2	111	ic	IS, inguinal LN, lower ext	Walking	Uptake percentage and clearance rate (rate constant)	Unknown	Unknown	China; Poland	[23]
1995	Freedman DO	^99m^Tc-labeled dextran	Unknown	Unknown	0.25	92.5	sc	IS, inguinal LN, lower ext	Feet	Uptake percentage, uptake counts per second, clearance rate (rate constant), and appearance time	45	Both	USA; Brazil	[24]
1996	Bull RH	^99m^Tc-antimony sulfide	Unknown	Unknown	Unknown	0.1	Unknown	IS, inguinal LN, axillary LN	Walking	Uptake percentage	120	Static	UK	[25]
1996	Harwood CA	^99m^Tc-antimony sulfide	Unknown	Unknown	Unknown	Unknown	sc	IS, inguinal LN, lower ext	None	Uptake percentage	120	Static	UK	[26]
1997	Pecking AP	^99m^Tc-Rhenium sulfide	Unknown	Unknown	Unknown	Unknown	Unknown	Unknown	None	Lymph velocity, clearance rate, clearance rate (rate constant), and half clearance time	Unknown	Unknown	France	[27]
1998	Bräutigam P	^99m^Tc-HSA	No	<80 nm	Unknown	20	sc	IS, inguinal LN	Cycling	Uptake percentage	120	Static	Germany	[28]
1999	Svensson W	^99m^Tc-HIG; ^99m^Tc-HSA	Unknown	Unknown	<0.3	35	ic	IS, inguinal LN, axillary LN, lower ext	None	Uptake counts per minute, clearance percentage, and blood appearance percentage	180	Both	UK	[29]
2001	Stanton AW	^99m^Tc-HIG	Unknown	Unknown	0.2	35	sc	IS, axillary LN, upper ext	Hand	Clearance rate (rate constant) and rate constant ratio (ksubcutis: kmuscle)	360	Both	UK	[30]
2001	Suga K	^99m^Tc-HSA	Unknown	4 nm	0.065	37	ic	lower ext	None	Relative increase in maximal activity (RIMA) and half clearance time	90	Both	Japan	[31]
2002	Pain SJ	^99m^Tc-HIG	Unknown	Unknown	0.2	3 and 1, respectively	sc	IS	Hand	Clearance rate (rate constant) and blood appearance percentage	180	Static	UK	[32]
2002	Szuba A	^99m^Tc-sulfur colloid	Filtered	<200 nm	0.2	9.25	sc	axillary LN, upper ext	Hand	Uptake ratio and dermal backflow ratio (DBR)	300	Static	USA	[33]
2003	Pain SJ	^99m^Tc-HIG	Unknown	Unknown	0.2	4 and 1, respectively	sc	IS	Hand	Clearance rate (rate constant), blood appearance rate (linear constant), and rate constant ratio (dividing krecovery by kdepot)	180	Static	UK	[34]
2003	Stanton AW	^99m^Tc-HIG	Unknown	Unknown	0.2	35	im	IS, upper ext, torso	None	Uptake percentage, clearance rate (rate constant), and rate constant ratio (kswollen:kcontralat)	300	Both	UK	[35]
2004	Gothard L	^99m^Tc-Nanocoll	Yes	Unknown	0.2	35	sc	IS	None	Clearance rate (rate constant)	180	Static	UK	[36]
2004	O’Mahony S	^99m^Tc-HIG; ^99m^Tc-HSA	Yes	Unknown	2	120	sc, ic	IS, heart, liver	None	Clearance rate (rate constant) and blood appearance rate (linear constant)	180	Dynamic	UK	[37]
2004	Pain SJ	^99m^Tc-HIG	Unknown	Unknown	0.2	2 and 1, respectively	sc	IS	Hand	Uptake percentage, clearance rate (rate constant), blood appearance rate (linear constant), rate constant ratio (dividing krecovery by kdepot), and uptake rate (linear constant)	180	Static	UK	[38]
2004	Pain SJ	^99m^Tc-HIG	Unknown	Unknown	0.2	2 and 1, respectively	sc	IS	Hand	Clearance rate (rate constant), blood appearance rate (linear constant), and rate constant ratio (dividing kaffected by kunaffected)	180	Static	UK	[39]
2004	Pain SJ	^99m^Tc-HIG	Unknown	Unknown	0.2	5 and 1, respectively	sc	IS	None	Clearance rate (rate constant) and blood appearance rate (linear constant)	180	Static	UK	[40]
2005	Modi S	^99m^Tc-HIG	Unknown	Unknown	0.2	0.56	epifascial	IS	None	Clearance rate (rate constant)	180	Static	UK	[41]
2006	Kafejian-Haddad AP	^99m^Tc-labeled dextran	Unknown	Unknown	0.3	185	ic	inguinal LN, popliteal LN, lower ext	None	Clearance percentage	180	Static	Brazil	[42]
2006	O’Mahony S	^99m^Tc-HIG	Unknown	Unknown	0.1	40	sc, ic	IS, upper ext	None	Uptake percentage, clearance rate (rate constant), and blood appearance rate (linear constant)	180	Static	UK	[43]
2006	Stanton AW	^99m^Tc-HIG	Unknown	Unknown	0.2	35	sc	IS, axillary LN, upper ext	None	Uptake percentage and clearance rate (rate constant)	300	Both	UK	[44]
2007	Szuba A	^99m^Tc-HSA	Yes	Unknown	Unknown	9.25	sc	IS, axillary LN	None	Uptake ratio, uptake counts per second, and clearance percentage	120	Static	Poland	[45]
2007	Celebioglu F	Unknown	Yes	<80 nm	1	10	sc	IS, axillary LN	None	Half clearance time, appearance time, and uptake ratio	180	Both	Sweden	[46]
2007	Lane KN	^99m^Tc-antimony sulfide	Unknown	Unknown	0.05	4.5	sc	IS, axillary LN, upper ext	Arm	Uptake percentage and clearance rate (rate constant)	65	Static	Canada	[47]
2007	Modi S	^99m^Tc-HIG	Unknown	Unknown	0.05	10	ic	axillary LN, upper ext	Hand	Uptake percentage, clearance rate (rate constant), blood appearance rate (linear constant), appearance time, Ppump (mmHg), and lymph velocity	120	Both	UK	[48]
2008	Dabrowski J	^99m^Tc-HSA	Yes	Unknown	Unknown	37	sc	inguinal LN	Feet	Uptake percentage and appearance time	120	Static	Poland	[49]
2008	Damstra RJ	^99m^Tc-HSA	Unknown	<80 nm	Unknown	40	sc	inguinal LN	Walking	Uptake percentage	120	Static	Netherlands	[50]
2008	Unno N	^99m^Tc-HSA	Unknown	Unknown	0.3	111	sc	inguinal LN, popliteal LN	None	Appearance time	20	Dynamic	Japan	[51]
2009	Haddad Filho D	^99m^Tc-dextran	Unknown	Unknown	Unknown	9.25	sc	lower ext	None	Clearance percentage and half clearance time	120	Both	Brazil	[52]
2009	Gebouský P	^99m^Tc-sulfur colloid; ^99m^Tc-HSA	Unknown	Unknown	0.1–0.2	20	sc	axillary LN, supraclavicular LN, upper ext	Arm	Radiotracer accumulation modeling with Bayesian estimates	180	Static	Czech Republic	[53]
2009	Stanton AW	^99m^Tc-HIG	Unknown	Unknown	0.2	0.6	sc, im	IS	None	Clearance rate (rate constant) and rate constant ratio (ksubcutis: kmuscle)	180	Static	UK	[54]
2010	Tartaglione G	^99m^Tc-HSA	Yes	Unknown	0.3–0.4	50	ic	inguinal LN, axillary LN	Multiple	appearance time	20	Static	Italy; UK	[55]
2012	Jensen MR	^99m^Tc-HSA	Unknown	Unknown	0.1	37	sc	IS	Cycling	Clearance rate (rate constant), blood appearance rate, and half clearance time	25	Dynamic	Denmark	[56]
2012	Kim YB	^99m^Tc-phytate	Filtered	Unknown	Unknown	148	sc	IS, inguinal LN, lower ext	Walking	Uptake ratio	120	Static	Korea	[57]
2014	Devoogdt N	^99m^Tc-HSA	Yes	Unknown	0.2	85	sc	IS, axillary LN	Hand	Uptake percentage, uptake rate (linear constant), clearance percentage, and appearance time	70	Both	Belgium	[58]
2015	Bains SK	^99m^Tc-HSA	Yes	Unknown	0.2	20	sc	axillary LN, upper ext	None	Uptake percentage and clearance rate (rate constant)	185	Static	UK	[59]
2015	Iimura T	^99m^Tc-phytate	Unknown	200–1000 nm	1	185	sc	inguinal LN, lower ext, liver, left ventricle	Pressure	Liver to blood ratio (LBR) and inguinal LN to blood ratio (ILBR)	90	Static	Japan	[60]
2015	Yoo JN	^99m^Tc-phytate	Unknown	Unknown	Unknown	185	sc	axillary LN, upper ext	Hand	Uptake percentage and uptake ratio	120	Static	Korea	[61]
2016	Cintolesi V	^99m^Tc-HSA	Yes	Unknown	0.05	8.4	ic	axillary LN, upper ext	None	Uptake percentage, Ppump (mmHg), lymph velocity, and maximum count	70	Both	UK	[62]
2016	Szuba A	^99m^Tc-HSA	Yes	Unknown	Unknown	9.25	sc	IS, axillary LN	None	Uptake ratio and clearance ratio (IS count time 0/time 2 h)	120	Static	Poland	[63]
2016	Zetterlund L	^99m^Tc-dextran; ^99m^Tc-HSA	Yes	Unknown	Unknown	20	sc	IS	None	Uptake percentage	180	Static	Sweden	[64]
2017	Kim P	^99m^Tc-phytate	Unknown	Unknown	Unknown	Unknown	sc	IS, axillary LN	Hand	Uptake percentage, uptake ratio, and clearance percentage	60	Static	Korea	[65]
2017	Sarri AJ	^99m^Tc-phytate	Unknown	Unknown	0.5	37	sc	axillary LN	None	Uptake rate (linear constant)	90	Both	Brazil	[66]
2017	Toyserkani NM	^99m^Tc-HSA	Unknown	Unknown	0.1	20	ic	IS, upper ext	None	Mean transit time	300	Both	Denmark	[67]
2018	Yoon JA	Unknown	Unknown	Unknown	Unknown	Unknown	Unknown	axillary LN, upper ext	Unknown	QAI	Unknown	Unknown	Korea	[68]
2018	Cousins E	^99m^Tc-HSA	Yes	25 nm	0.2	25	sc	IS, inguinal LN, axillary LN	None	Uptake percentage	120	Both	UK	[69]
2018	Hvidsten S	^99m^Tc-HSA	Unknown	Unknown	0.1	20	ic	IS, upper ext	None	Mean transit time, clearance rate (rate constant), and retention function best model fit constants (k, a, and b)	300	Both	Denmark	[70]
2018	Keramida G	^99m^Tc-HSA	Yes	Unknown	0.1	20	sc	IS, inguinal LN	None	Uptake percentage, clearance rate (rate constant), and lymphatic drainage efficiency LDE (%) = 100(IIQ/k)	150	Static	UK	[71]
2019	Hou G	^68^Ga-NOTA-Evans Blue (NEB)	Unknown	Unknown	0.5	37	sc	inguinal LN, lower ext	None	SUVR	40	Static	China; USA	[72]
2019	Kim YH	^99m^Tc-phytate	Unknown	Unknown	Unknown	148	sc	IS, axillary LN, supraclavicular LN, upper ext	Hand	Uptake ratio	120	Static	Korea	[73]
2019	Nganga EC	^99m^Tc-HSA	Yes	Unknown	Unknown	20–40	ic	IS, inguinal LN	Walking	Uptake percentage	90	Static	Kenya	[74]
2019	Sarica M	^99m^Tc-HSA	No	Unknown	0.2	25	sc	inguinal LN	None	Uptake percentage and clearance percentage	120	Static	UK	[75]
2019	Toyserkani NM	^99m^Tc-HSA	Unknown	Unknown	0.1	20	ic	IS, upper ext	None	Mean transit time	300	Static	Denmark	[76]
2020	Kim J	^99m^Tc-phytate	Filtered	100 nm	0.2	37	sc	axillary LN, supraclavicular LN, upper ext	Hand	Uptake percentage and uptake ratio	120	Static	Korea	[77]
2020	Kim K	^99m^Tc-phytate	Unknown	Unknown	0.1–0.2	37	sc	axillary LN, upper ext	Multiple	Uptake percentage and uptake ratio	60	Static	Korea	[78]
2020	van de Pas CB	Unknown	Unknown	Unknown	1	Unknown	sc	IS, inguinal LN	None	Uptake percentage and clearance percentage	120	Static	Netherlands	[79]
2021	Kwon HR	^99m^Tc-tin colloid; ^99m^Tc-phytate	Unknown	Unknown	0.1	37	Unknown	IS, inguinal LN, lower ext	Walking	Uptake percentage and clearance percentage	120	Static	Korea	[80]
2021	Sampathirao N	^99m^Tc-antimony sulfide	Unknown	Unknown	0.2	18.5	ic	IS, inguinal LN	Feet	Uptake percentage	180	Static	India	[81]
2022	Koike T	^99m^Tc-HSA; ^99m^Tc-phytate	Unknown	HSA (2–3 nm); phytate (100–1000 nm)	0.2	40	sc	inguinal LN	None	Uptake percentage and contrast-to-noise ratio (CNR) of DBF	120	Static	Japan	[82]
2023	McMeekin HJ	^99m^Tc-HSA	Yes	Unknown	Unknown	Unknown	sc	inguinal LN	None	Uptake percentage	150	Static	UK	[83]
2024	Myung Y	^99m^Tc-phytate	Unknown	Unknown	Unknown	37	sc	axillary LN, upper ext	None	Uptake percentage and clearance rate (rate constant)	60	Static	Korea	[84]

^ ic, intracutaneous; sc, subcutaneous; im, intramuscular;

* from the time of injection to the last acquired measurement.

**Table 2.** The calculations quantifying lymphedema during lymphoscintigraphy.

Quantification Method	Working Definition	Number of Studies
Uptake percentage	The % of total injected activity within an ROI at a specific time point.	37
Clearance rate (rate constant)	The slope of the linear plot of counts remaining in the injection depot vs. time.	24
Uptake ratio	Any ratio between the counts of two ROIs at a single time point (this includes the quantitative asymmetry index (QAI) and a comparison between the ROIs of the affected and unaffected sides).	12
Clearance percentage	The % of total injected activity that remains at the injection depot at a specific time point.	9
Appearance time	The time until a specified SUV is reached within an ROI (usually recorded when the SUV of an ROI reaches half of its maximum value).	8
Uptake counts per time	The corrected counts within an ROI or unit volume during a single time point (i.e., the counts obtained during a 1 s acquisition).	7
Rate constant ratio	The ratio between the rate constants of two ROIs (i.e., affected and unaffected sides; subcutis and muscle).	7
Blood appearance rate (linear constant)	The slope of the linear plot of the blood appearance percentage vs. time.	7
Half clearance time	The time until half of the initial counts recorded at the injection depot is removed.	5
Uptake rate (linear constant)	The slope of the linear plot of counts accumulated vs. time.	3
Mean transit time (MTT)	The time activity curves from each ROI are obtained, and the background and decay are corrected. Then, a best-fit retention function model is obtained based on the measured time activity curve and the input function (least squares regression method). The area under the retention function curve corresponds to the MTT.	3
Blood appearance percentage	The % of administered activity per liter of blood.	2
Model constants	A best-fit model is developed based on the time-activity curves from an ROI; the model constants are compared between patients.	2
Contrast-to-noise ratio (CNR) of DBF	The mean background counts is subtracted from the mean SUV of the area of dermal backflow; the result is divided by the standard deviation of the background counts (mean (ROI DBF) − mean (ROI BG))/(SD (ROI BG)).	1
Factorial analysis	Involves the software processing of dynamic lymphoscintigraphy studies developed to eliminate the need to select ROIs manually. The software extracts ROIs that have activity curves with the same temporal behavior.	1
Maximum count	The maximum counts recorded within an ROI during the study	1
Lymphatic drainage efficiency (LDE)	The % injected activity (IIQ) in ilio-inguinal nodes is divided by the % activity leaving the injection depot by 150 min (k) LDE (%) = 100 × (IIQ/k).	1

## Data Availability

No new data were created or analyzed in this study. Data sharing does not apply to this article.
